# Germline mutations in pancreatic cancer and potential new therapeutic options

**DOI:** 10.18632/oncotarget.17291

**Published:** 2017-04-20

**Authors:** Rille Pihlak, Juan W. Valle, Mairéad G. McNamara

**Affiliations:** ^1^ Division of Molecular and Clinical Cancer Sciences, University of Manchester, Manchester, United Kingdom; ^2^ Department of Medical Oncology, The Christie NHS Foundation Trust, Manchester, United Kingdom

**Keywords:** pancreatic cancer, germline mutations, BRCA1, BRCA2, PARP inhibitors

## Abstract

Due to short-lived treatment responses in unresectable disease, pancreatic ductal adenocarcinoma (PDAC) continues to be one of the deadliest cancers. There is availability of new information about germline and sporadic mutations in the deoxyribonucleic acid (DNA) damage repair pathway in PDAC in recent decades and the expectation is that novel targeted therapies will thus be developed. A variety of germline mutations (*BRCA2*, BRCA*1*, *PALB*2, *CDKN2A*, *ATM*, *TP53* and mismatch repair genes *MLH1*, *MSH2*, *MSH6*) have been reported in these patients with the highest prevalence being *BRCA1*/2. Positive results have been reported with the use of targeted therapies, particularly poly (ADP-ribose) polymerase inhibitors in *BRCA*-mutated ovarian and breast cancers, and their use is currently being investigated in germline-mutated pancreatic cancer. The aim of this review is to provide an outline of germline DNA damage repair mutations in pancreatic cancer and their effect on the incidence, outcomes and responses to different therapeutic options.

## INTRODUCTION

Pancreatic cancer is the 10th most common cancer in the UK (2013) and 12th most common cancer in the US [1, 2]. Unfortunately, pancreatic ductal adenocarcinoma (PDAC) has also been shown to be the most lethal human malignancy with the worst 5-year overall survival (OS) compared to other types of cancer [3]. The 5-year OS of all stages is around 7.7% in the US [1], and 3% in England and Wales (2010–2011) [2]. Even for patients who have had potentially curative surgery who receive adjuvant chemotherapy (gemcitabine and capecitabine), the 5 year overall survival is 28.8% according to the most recently-published data from the phase III randomised ESPAC-4 trial [4]. The longest overall survival reported for patients with metastatic disease was in the ACCORD trial where patients receiving the oxaliplatin, irinotecan, 5-fluorouracil and leucovorin combination (FOLFIRINOX) had a median overall survival of 11.1 months [5]. In the MPACT trial, the median OS for patients who received gemcitabine and nab-paclitaxel was 8.5 months [6]. In both of these studies, there are hints of possible subsets of patients that may be deriving significant benefit from the treatment, with tails observed in the Kaplan-Meier curves, and better characterisation of these patient subgroups is necessary to guide future therapeutic options. Sadly, the majority of clinical trials recruiting patients with advanced pancreatic cancer over the past 5 years have failed to demonstrate a more significant clinically meaningful benefit [7].

Due to short-lived treatment responses, pancreatic cancer is the 3rd and 5th most common cause of cancer death in the US and UK (2012) respectively, accounting for more than 5% of all deaths from cancer [1, 2], and is projected to become the second leading cause of cancer-related death by 2030 [8].

Most of the cases of pancreatic adenocarcinoma are thought to be sporadic, however approximately 5% to 10% occur in the presence of a family history of the disease [9].

Multiple syndromes and diseases [10–12] have been associated with an increased risk of developing pancreatic cancer, including familial atypical multiple mole melanoma (FAMMM) [13, 14], Peutz-Jeghers syndrome (PJS) [15, 16], hereditary pancreatitis [17], hereditary nonpolyposis colorectal carcinoma (HNPCC) [18], hereditary breast and ovarian cancer (HBOC) [19], and familial adenomatous polyposis [20, 21]. Although the numbers are small, the most common germline mutations in pancreatic cancer related to these syndromes are breast cancer 2 (BRCA2), breast cancer 1 (*BRCA*1), partner and localiser of BRCA2 (*PALB2*), cyclin-dependent kinase inhibitor 2A (*CDKN2A*), ataxia telangiectasia mutated (*ATM*), tumour protein p53 (*TP53*) and mismatch repair genes mutL homolog 1 (*MLH1)*, mutS homolog 2 (*MSH2)* and mutS homolog 6 (*MSH6)*.

Germline mutations, particularly in *BRCA*1 or *BRCA*2 cause a deficiency in deoxyribonucleic acid (DNA) damage repair (DDR) due to inhibition of repair of DNA double-strand breaks by the mechanism of homologous recombination [22].

Deoxyribonucleic acid repair has two different roles in cancer cells. Firstly, as in any other cell, cancer cells rely on DNA repair to survive the damage induced by genotoxic stress; and secondly, DNA repair enables cancer cells to accumulate genomic alterations that contribute to their aggressive phenotype [23]. Deoxyribonucleic acid damage repair mutations lead to chromosomal instability and tumorigenesis, through lack of repair or mis-repair of DNA damage [24] and BRCA1 functions in the signalling of DNA damage and its repair by homologous recombination and nucleotide-excision repair. The BRCA2 function has a more specific role in DNA repair, regulating the activity of RAD51, which is required for homologous recombination [25].

In recent years, there is new hope for patients with germline-mutated ovarian, breast and pancreatic cancer with the availability of poly (ADP-ribose) polymerase (PARP) inhibitors [26–28] and chemotherapeutic agents that induce DNA damage in the presence of impaired DNA repair.

In this review, the effects of germline DNA damage repair mutations are examined on the incidence, outcomes and responses to different therapeutic agents in patients with pancreatic cancer.

### Importance of family history and accuracy of current screening guidelines

The role that family history plays in germline-mutated pancreatic cancer has been investigated in many studies. Familial pancreatic cancer (FPC) is characterised by pancreatic cancer reported in at least 1 of a patient's first degree relatives (FDRs), in addition to their own diagnosis or families with ≥ 2 FDRs with PDAC [29].

It has been estimated that in the patient groups with familial pancreatic cancer, *BRCA*2 is the most common germline mutation, accounting for as many as 17% of FPC kindreds [30].

In the National Comprehensive Cancer Network (NCCN) Genetic/Familial high-risk assessment, breast and ovarian cancer screening guidelines (version 2.2016) [31], *BRCA* testing is advised in patients with PDAC if they have ≥ 1 first-, second- or third-degree blood relative with ovarian carcinoma at any age; or breast cancer ≤ 50 years of age; or two relatives with breast, pancreatic or prostate cancer (Gleason score ≥ 7) at any age; or any patient with PDAC who has Ashkenazi Jewish (AJ) ancestry.

It was hoped that with the availability of screening protocols, one could limit the number needed to be tested in order to identify mutations, but unfortunately studies have reported conflicting results, so family history or guidelines may not always predict mutational status.

In their study of 306 consecutive unselected patients with pancreatic cancer in Ontario, Canada, Holter et al. [32, 33] did not identify a statistically significant correlation of *BRCA*-mutation status with personal history of cancer, family history of PDAC, or family history of breast or ovarian cancer although there was a trend towards significant correlation with past history of cancer and family history of breast or ovarian cancer. Interestingly, none of the *BRCA*-mutation carriers identified met the criteria for familial PDAC. Thus, the majority of these patients with *BRCA* mutations would not have met the NCCN or the Ontario Ministry of Health and Long-Term Care, *BRCA*1 and *BRCA*2 genetic testing criteria.

In their other study, Grant et al. [34], aimed to improve the *BRCA* mutation prevalence estimation by selecting patients from three different groups based on family history of breast and/or ovarian cancer, pancreatic cancer, or neither. A significant association was reported between germline mutations and previous breast cancer in the proband or a first-degree relative (10.7% vs. 2.1%), and an additional significant change not based on the subgroups was found in colorectal cancer in the proband or a first-degree relative (11.1% vs. 2.8%). Interestingly, no association was reported between mutation carrier status and first-degree relatives with PDAC, age at diagnosis, or stage at diagnosis.

In the study by Salo-Mullen et al. [9] (159 patients with PDAC who pursued genetic testing), 22.9% met the criteria for FPC. Fifty-six patients were classified as having a “very strong” family history of cancer (2 close relatives among first degree relatives or second degree relatives [SDRs] with a *BRCA*-associated cancer), and the mutation prevalence in that group was 16.7% among the AJ patients (30 patients) and 7.7% among the non-AJ patients (26 patients). Mutational prevalence was 15.8% and 7.4% among AJ patients with either “weak” family history (1 close relative with a *BRCA*-associated cancer) or PDAC only in the proband and 11.1% in the non-AJ patients with weak family history and PDAC. The NCCN guidelines have changed over the years, therefore the percentage of patients with mutations meeting the requirements for screening changed from 51% in 2013 to 73.5% in 2014 and 93.4% in the 2015 NCCN guidelines. The major cause of this discrepancy was the initiation of testing of all AJ patients with PDAC as opposed to those only having at least 1 other family member with a *BRCA*-associated malignancy [9].

In an Italian study, there was a strong correlation between familial pancreatic cancer and the *CDKN2A* mutation, as 5 out of 16 patients (31%) with FPC carried the mutation (225 patients with PDAC enrolled) [35]. These findings suggest that a sizeable subset of Italian FPC families may carry a *CDKN2A* mutation [35] which has not been demonstrated in any other region.

In 2007, Couch et al. [36] analysed affected probands from 151 high-risk families for the *BRCA*2 mutation and identified five mutations (3 in families with ≥ 2 first-degree relatives with PDAC, 2 in families with ≥ 2 second-degree relatives with PDAC). In their high-risk pancreatic cancer families, 3% carried *BRCA*2 mutations. Together with their previous study [30], they estimated that *BRCA*2 mutations accounted for 6% of pancreatic cancers in high-risk families for pancreatic cancer and 6% of families fulfilled the criteria of familial pancreatic cancer. So the prevalence of the *BRCA*2 mutation can be as high as 15% in families with many first or second-degree relatives with PDAC and as low as 3% in high-risk families with low numbers of PDAC.

Other screening guidelines like the American College of Gastroenterology Clinical Guideline: Genetic Testing and Management of Hereditary Gastrointestinal Cancer Syndromes [37] and the International Cancer of the Pancreas Screening (CAPS) Consortium summit on the management of patients with increased risk for familial pancreatic cancer [38] are in use, but they haven't been evaluated extensively in clinical trial settings.

### Age of PDAC onset in patients with *BRCA* mutations

Whether germline mutations in pancreatic cancer have a link with the young onset of PDAC has been debated in many studies. In a remarkable case of “Family X” which had autosomal dominantly-inherited pancreatic cancer (four generations with 18 cases of PDAC [*n* = 9] or pre-cancerous Pancreatic Intraepithelial Neoplasia [PanIN] 2/3 [*n* = 9]) [39], successive generations of affected families with familial PDAC developed PDAC significantly earlier than previous generations, resulting in the phenomenon of ‘genetic anticipation’ [10, 40]. In the European study of 1223 at-risk individuals for PDAC [41] (106 families with 264 affected individuals), there were 80 affected child-parent pairs and the children died at a median of 10 years earlier than the parents. The median age of death from PDAC was 70, 64 and 49 years for the three generations, respectively. The same was reported in a German national case collection for familial pancreatic cancer [42]. In the study by Salo-Mullen et al, it was reported that the mean age at the time of diagnosis was significantly younger in all mutation carriers (58.5 years) compared to those not carrying the mutation (64 years) [9]. However, in other studies the carrier status was not significantly associated with age at diagnosis [33, 34, 43]. Therefore, the correlation between germline mutations and young onset PDAC remains unspecified due to these conflicting results and international consensus documents do not recommend screening of high risk individuals before the age of 50 with the exception of PJS and hereditary pancreatitis [38, 44].

Current European pancreatic cancer screening programmes include families with ≥ 2 patients with PDAC, or presence of Lynch syndrome and 1 patient with PDAC, melanoma and 1 patient with PDAC, Peutz–Jeghers syndrome, hereditary pancreatitis and families with one early-onset PDAC (< 50 years) [29, 45]. Unfortunately these studies demonstrate that there is no clear guidance on how to screen for germline mutations in PDAC, although there are clear high-risk groups where mutation testing is advised. Based on the observational studies of high risk groups and the unknown magnitude of correlation between family history and PDAC risk, mutational screening of patients with young onset of PDAC should be discussed with a genetics team.

### Development of PDAC in patients with previously known germline mutations

In patients with previously-identified germline mutations, the risk of developing pancreatic cancer is increased. It has been reported that germline mutations in *BRCA*1 and *BRCA*2 predispose to pancreatic cancer [46, 47].

In the largest follow-up study of *BRCA*-mutated patients in the Hereditary Breast Cancer Study Group, eight new cases of pancreatic cancer were identified, out of 5089 women, in the database of *BRCA*1 and *BRCA*2 carriers, versus 3.28 expected pancreatic cancers. The *BRCA*1 and *BRCA*2 carrier standardised incidence ratio (SIR) was 2.55 and 2.13, respectively. The 5-year survival rate was 5% for *BRCA*1 mutation carriers and 4% for *BRCA*2 [46]. For women above the age of 50, the annual incidence rate was 37 per 100 000 per year for *BRCA*1 carriers and 39 per 100 000 per year for *BRCA*2 carriers [46], compared to 11 per 100 000 women in age-adjusted historical controls [1].

The most recent pancreatic cancer-specific surveillance study for high-risk people described surveillance data for 411 asymptomatic high-risk individuals who participated in the programmes in three European expert centres (Germany, The Netherlands and Spain). The study included 178 *CDKN2A* mutation carriers, 214 individuals with familial pancreatic cancer, and 19 *BRCA*1/2 or *PALB2* mutation carriers. Among 178 *CDKN2A* mutation carriers, PDAC was found in 7.3% (*N* = 13), 0.9% in the familial pancreatic cancer group (*N* = 2; 1 advanced PDAC, 1 grade 2 neuroendocrine tumour [NET]) and 1 PDAC was diagnosed among *BRCA*2 carriers. The resection rate of PDAC for *CDKN2A* mutation carriers was 75%, and the 5-year survival rate 24%. As this resection rate and the survival data at 5 years was better than historical controls at that time (before the ESPAC-4 data [4]), this study demonstrated that surveillance of *CDKN2A* mutation carriers was relatively successful with different combinations of magnetic resonance imaging (MRI), endoscopic ultrasound (EUS) and magnetic resonance cholangiopancreatography (MRCP) [48] in the context of this trial, detecting most PDACs at a resectable stage. As the numbers were very small for families with FPC, the benefit of their surveillance is still unknown [29] and equally the growth pattern differences between *CDKN2A* mutated and FPC cancers is unknown, so the effect that this could have on surveillance strategies remains to be seen.

### Germline mutations among patients with PDAC

Over the past 3 decades, multiple studies have looked at the number of different germline mutations in patients with PDAC (Table 1). Initial studies focused solely on *BRCA*2 mutations, but have now evolved into whole genome sequencing (WGS) of tumours in patients with PDAC [49]. In Table 1, nine studies are included where different germline mutations were assessed in specific cohorts of patients with PDAC. These studies are not directly comparable as some have looked at consecutive patients with PDACs, some have limited the mutation testing to only FPC, patients pursuing genetic testing or those of Ashkenazi Jewish origin, and some have only analysed resected tumours.

**Table 1:** Germline mutations in pancreatic ductal adenocarcinoma

	Technique	Stages	*ATM (N)*	*BRCA1 (N)*	*BRCA2 (N)*	*MLH1 (N)*	*MSH2 (N)*	*MSH6 (N)*	*TP53 (N)*	*PALB2 (N)*	*CDKN2*A/p16 (*N*)	*PALLD (N)*	*FANCC (N)*
***Grant et al. 2015* (*N* = 290)** [34]	Blood Tissue NGS, IHC	I–IV NS	3	1	2	1	2	1	1	0	0	-	-
***Holter et al. 2015* (*N* = 306)** [33]	Blood DS, LRA	I–II 75 III–IV 231	-	3	11	-	-	-	-	-	-	-	-
***Salo-Mullen et al. 2015* (*N* = 159)**^a^ [9]	Blood Tissue FMA FGS LRA IHC	N/K	-	4	13	1/36	2/36	1/36	-	1/48	2/17 p16	-	-
***Waddell et al. 2015* (*N* = 100)** [51]	Tissue WGS	N/K	NS	2 S	7 (4G, 3S)	NS	NS	-	74	3	35	-	-
***Kim et al 2014.* (*N* = 555)** [53]	Tissue IHC SS	R only I–II 36 III–IV 476	67	-	-	-	-	-	-	-	-	-	-
***Ghiorzo et al. 2012* (*N* = 225)** [35]	Blood DS MLPA	I–II– 63 III–IV 153 N/K– 9	-	1/16	0/16	-	-	-	-	0/16	5, 7% *CDKN2A*	0/16	-
***Ferrone et al. 2009* (*N* = 145)**^b^ [43]	Tissue FMA	R only I–II 142 III–IV 3	-	2	6	-	-	-	-	-	-	-	-
***Couch et al. 2005* (*N* = 421/389)**^c^ [56]	Blood tissue dHPLC, LOH	N/K	-	-	-	-	-	-	-	-	-	-	2
***Lal et al. 2000* (*N* = 102/38)**^d^ [19]	Blood Tissue FMA PFT, IHC	N/K	-	1/7^e^	3/38	0/3^f^	0/3^f^	-	-	-	1/38	-	-

^a^Subset of patients who pursued genetic testing.

^b^Only Jewish patients with PDAC.

^c^Out of 421 patients, 389 had pancreatic adenocarcinoma.

^d^38 patients were characterised as high or intermediate risk for development of pancreas cancer and were screened for mutations.

^e^BRCA1 testing was confined to patients with family histories suggestive of breast-ovarian cancer syndrome.

^f^Mismatch repair gene expression was analysed only in patients with a personal or family history suggestive of HNPCC and with adequate tumour and normal tissue for immunohistochemistry.

Abbreviations: *ATM*- Ataxia telangiectasia mutated; *BRCA1*- breast cancer 1; *BRCA2*- breast cancer 2; *MLH1*- mutL homolog 1; *MSH2*- mutS homolog 2; *MSH6*- mutS homolog 6; *TP53*- tumour protein p53; *PALB2*- partner and localiser of BRCA2; *CDKN2A/p16*- cyclin-dependent kinase inhibitor 2A ; *PALLD*- palladin, cytoskeletal associated protein;*FANCC*- Fanconi anaemia complementation group C.

DS- direct sequencing; NGS- next generation sequencing; WGS- whole genome sequencing; FGS- full gene sequencing; LRA- large rearrangement analysis; IHC- immunohistochemical staining analysis for the DNA mismatch repair proteins; MLPA- Multiplex ligation-dependent probe amplification analysis; PFT- Protein function test; FMA- founder mutation analysis; SS- Sanger sequencing; dHPLC- WAVE denaturing high performance liquid chromatography analysis; LOH- loss of heterozygosity analysis. R- Resected; N/K- Not known; NS- Not specified; TMA- Tissue microarrays; G-germline mutations; S-somatic mutations.

Comparisons between germline-mutated pancreatic cancer studies are also complicated by variations in methodology of mutational analyses, as earlier studies may have focused on specific mutations in tumours, with more recent studies sequencing the whole genome.

Identification of *BRCA* mutations are generally standardised by founder mutation analysis, full gene sequencing and large rearrangement analysis, but studies discussed in this review do not incorporate all of these techniques consistently.

In the study by Grant et al. [34], 290 patients with pancreatic cancer were selected from three different groups according to family history; 71 from 136 Ontario Pancreas Cancer Study (OPCS) patients with a family history of PDAC, 39 from 85 patients with a family history of breast or ovarian cancer but no family history of PDAC, and 180 from 487 OPCS patients without a family history of pancreatic, breast, or ovarian cancer. Among these 290 patients, 11 mutations were discovered; 3 in *ATM*, 1 in *BRCA*1, 2 in *BRCA*2, 1 in *MLH1*, 2 in *MSH2*, 1 in *MSH6*, and 1 in *TP53*. The prevalence of mutations in all 13 genes was 3.8%. Interestingly, their cohort identified no *PALB2* or *CDKN2A* mutation carriers.

A second study by the same group described 306 unselected, consecutive, incidental patients with PDAC at a single centre over a 2-year period. Among 14 patients (4.6%) with *BRCA* mutations identified, 11 had a *BRCA*2 mutation and 3 had a *BRCA*1 mutation [33]. In an older study published in 2000 by the same authors [19], patients included had pancreatic cancer at high- or intermediate-risk of mutational status and 38 out of 102 (37%) patients were characterised as such. Five cases of germline mutations were identified (13%) in this group (p16 = 1; *BRCA*1 = 1; *BRCA*2 = 3) and all the patients with *BRCA*1 and *BRCA*2 mutations were of Ashkenazi Jewish inheritance.

The Italian study performed by the Genoa Pancreatic Cancer Study Group reported conflicting results. Tumours of 225 consecutively-enrolled patients with PDAC were tested for *CDKN2A* mutations. A subset of the patients was classified as having FPC, and only they were also tested for other mutations in *PALLD*, *PALB2*, *BRCA*1 and *BRCA*2, as FPC candidate genes. Only 5.7% of the patients were found to have *CDKN2A* mutations and none of the patients classified as having FPC harboured germline mutations in *PALLD*, *PALB2* or *BRCA*2. One family had a *BRCA*1 mutation [35].

This data provides evidence that, in different countries, the prevalence of mutations may differ greatly, as *CDKN2A* mutations account for smaller numbers, as in some Canadian studies, for example [19, 34]. The patients in the Genoa study [35] were all treated in Italy but there is no data about the ethnicity of the group or whether lack of Jewish heritage played a role in the low mutational status.

### Prevalence of germline mutations in potential high-risk groups

As already discussed, family history of cancer or Jewish heritage can predispose to pancreatic cancer and this has been investigated in many studies, two of which are discussed here.

In the previously-mentioned study by Salo-Mullen et al., with the preselected group of 159 patients with PDAC, who pursued genetic testing, the authors also recorded the mutational status in the whole cohort in addition to assessing for mutational prevalence in weak and strong family history groups. Among all 159 patients, they reported 24 pathogenic mutations (15.1%), including *BRCA*2 (13 mutations), *BRCA*1 (4 mutations), p16 (2 mutations), *PALB2* (1 mutation), and Lynch syndrome (4 mutations). Among AJ patients (95 patients), *BRCA*1/*BRCA*2 mutations were found in 13.7% and in 7.1% of non-AJ patients (56 patients) [9]. As these patients pursued genetic testing, their family history predisposed to mutations, and thus the likelihood of detection was also greater. The mutational prevalence of 15.1% is similar to the publication by Lal et al. where 13% was reported [19].

In Ferrone et al. [43], patients with resected pancreatic cancer reviewed retrospectively were included who self-declared as Jewish (all were assumed to be of AJ ethnicity). Among the 145 patients included, 8 patients (5.5%) were *BRCA* founder mutation positive (*BRCA*1 = 2, *BRCA*2 = 6). These data were also compared to those from control patients who were cancer-free and of AJ origin in the Washington DC area, where the mutation was identified in significantly lower numbers of people; 1.1%. The study only looked at specific AJ founder mutations and not at any other possible mutations in *BRCA*1 or *BRCA*2, therefore the relatively small number of mutations in this high risk group of patients (AJ) is difficult to interpret.

### Deeper, whole exome-or genome sequencing of PDAC samples

Recent years have seen a rapid reduction in the cost of genomic sequencing with identification of a variety of new potential molecular targets for therapy [50]. However, the rate that genomic data has accumulated has raised concerns that it may not be possible to interpret properly, adequately capture, or fully analyse [50], as deeper sequencing may identify mutations that are not clinically significant.

One of the most ground-breaking studies on PDAC mutational status in recent years was the Waddell et al. [51] study where WGS and copy number variation (CNV) analysis was performed on 100 resected PDAC samples. According to these findings, PDAC may be classified into four subtypes based on structural variation profiles, implicating molecular mechanisms underlying some of these events with potential clinical relevance. The four subtypes reported were; stable subtype, locally-rearranged subtype, scattered subtype and unstable subtype.

Ten out of 14 unstable tumours fell within the top quintile of the *BRCA* signature which was associated with deleterious mutations of *BRCA*1 (*n* = 2), *BRCA*2 (*n* = 7), and *PALB2* (*n* = 2). Out of 7 mutations in *BRCA*2, 4 were germline in origin and 3 had a somatic mutation. Both *BRCA*1 mutations were somatic [51].

It was also reported that combining structural variation events with deleterious point mutations increased the prevalence of inactivation events. This was best seen in relation to *TP53*, where 3 structural variants and 71 mutations were found. In relation to SMAD4, 9 structural variants and 22 mutations were identified and in relation to *CDKN2A*, there were 11 structural variants and 24 mutations. Two new genes, *KDM6A* and *PREX2*, had pathogenic mutations and structural variants in ≥ 10%, and these had not been previously described in human PDAC [51]. In the more recent study, published in 2016, the same group performed WGS and deep-exome sequencing with gene copy number analysis on 456 samples from patients with PDAC and their histopathological variants. They reported germline mutations in 5% and somatic mutations in 12% in the *BRCA* pathway (*BRCA*1, *BRCA*2, *ATM* and *PALB2*). They further categorised their results to describe four pancreatic cancer subtypes: squamous, pancreatic progenitor, immunogenic and aberrantly differentiated endocrine exocrine (ADEX) [52], and although these subtypes may not have clinical relevance at this time, they may guide treatment decisions in the future.

### Identification of additional mutations in PDAC samples

Deleterious *ATM* mutations have also been recognised in the germline of families with FPC. Kim et al. [53] examined tumoural *ATM* loss among 397 surgically-resected patients with PDAC and observed *ATM* loss in one cancer known to have bi-allelic inactivation of *ATM* and in 50 others of the first 396 (12.8%) cases. Although they only looked at samples from patients who had undergone surgery, there were still low numbers of stage I–II disease (*N* = 36), and most of the patients had advanced disease (stage III–IV [*N* = 476]). Loss of *ATM* was discovered significantly more often in patients with a family history of pancreatic cancer (12/49; 24.5%) than in those without (38/347; 11.0%). In another study, Roberts et al. [54] used next-generation sequencing (NGS), including whole-genome and whole-exome analyses, and identified heterozygous, constitutional *ATM* gene mutations in 2 kindreds with familial pancreatic cancer. Among severely-affected families with 3 or more pancreatic cancer cases, 4 deleterious mutations were found in 87 families (*P* = 0.009). In the study by Grant et al. [34], *ATM* mutations were only found in 3 out of 290 patients (1%) tested.

Biankin et al. [55] identified *ATM* mutations in a significant proportion of patients (8%) in their exome sequencing and copy number analysis of 99 early stage sporadic pancreatic ductal adenocarcinoma samples, demonstrating the importance of *BRCA*-mediated DNA damage repair mechanisms both in sporadic and familial disease. Roberts et al. demonstrated that among familial pancreatic cancer probands, 4/166 (2.4%) carried deleterious *ATM* mutations and the numbers were even higher (4.6%) in families with more than 3 affected members [54]. These studies exhibited that *ATM* loss plays a role in pancreatic cancer but the clinical significance of this aberration is still unknown, as there are no trials that have targeted this specific mutation in patients with PDAC.

Some studies also imply that Fanconi genes, in addition to *BRCA*2, play a role in inherited forms of pancreatic cancer. Couch et al. [56] reported that amongst patients with young-onset PDAC (< 55 years), with no family history of PDAC, two truncating *FANCC* mutations were identified, but no truncating *FANCG* mutations. Both mutations were associated with loss of heterozygosity of the wild-type allele in the corresponding pancreatic tumours. Their data support the idea that inherited mutations in *FANCC* can predispose to pancreatic cancer, although the numbers of these mutations might be very small compared to other germline mutations.

The role of hereditary nonpolyposis colorectal cancer or Lynch syndrome in pancreatic cancer is still widely debated. The most common form of hereditary colon cancer is hereditary nonpolyposis colorectal cancer, which is mainly caused by mutations in mismatch repair genes; *MSH2 or MLH1*, and more rarely by mutations in *MSH6, PMS1* and *PMS2* [10]. The role of mismatch gene variants in pancreatic cancer is still not clear. Dong et al. retrospectively reviewed 706 patients with PDAC and 706 cancer-free controls, and genotyped 102 single-nucleotide polymorphisms (SNPs) of 13 mismatch repair-related genes using the mass spectroscopy-based MassArray method. They found that haplotypes of O6-methylguanine-DNA methyltransferase (*MGMT*), *MSH6*, *PMS1* Homolog 2 (*PMS2*), *PMS2*-like 3 (*PMS2L3*), and tumour protein 73 (*TP73*) were significantly associated with an increased pancreatic cancer risk (*P* = 0.0015). This suggests that mismatch repair gene variants may affect susceptibility to pancreatic cancer but the magnitude is still unknown [21].

Another much discussed [10] mutation in familial pancreatic cancer is the Palladin mutation (*PALLD*) which was shown to be the main mutation in the previously mentioned exceptional family, Family X [39], in which pancreatic adenocarcinoma was inherited in an autosomal dominant fashion with high penetrance. Four generations of Family X included 18 cases of either adenocarcinoma (*n* = 9) or histologically-proven precancerous PanIN 2 and 3 (*n* = 9). Thirty five family members were genotyped [57] and mutations were found in the *palladin* gene. However, no link between pancreatic cancer and *PALLD* mutation has been found in subsequent studies [58, 59]. Many indicate that the mutational prevalence in patients with pancreatic cancer is very low [35].

### *BRCA*ness in pancreatic cancer

In current clinical trials (Table 2), mutational status is based on identification of a germline mutation in *BRCA*1 or *BRCA* 2. However, in addition to germline mutations, sporadic mutations can also result in a so called *BRCA*ness phenotype. Turner et al. define *BRCA*ness in their publication as “traits that usually occur in *BRCA*1/2 mutation carriers but are also present in some sporadic cancers”. In addition, *BRCA*ness encompasses separate sets of features, reflecting the distinct consequences of mutations in *BRCA*1, *BRCA*2, genes involved in Fanconi anaemia or other genes [22]. An alternative explanation is that *BRCA*ness exists when a DDR defect is present in a tumour in the absence of a germline *BRCA*1 or *BRCA*2 mutation [60]. The concept of BRCAness as a homologous recombination deficiency (HRD) has been explored in two recent clinical trials [61, 62] in patients with ovarian cancers as a marker for potential response to PARP inhibitors in non-germline BRCA-mutated tumours. Both of these trials have reported positive responses to PARP inhibitors in BRCA-wildtype, but HRD-high tumours, highlighting the importance of evaluating this in other tumour groups.

**Table 2:** Ongoing clinical trials in germline-mutated pancreatic cancer registered on Clinicaltrials.gov

Trial ID		Phase	Number of patients	Status of trial	Countries involved
**NCT01296763**[85]	A Randomised Multi-centre Phase I/II Trial of Irinotecan, Cisplatin, Mitomycin C (ICM) with or without olaparib (AZD2281) in Patients With Advanced Pancreatic Cancer	I	18	Completed, no phase II	US
**NCT01585805** [67]	A Randomised Phase II Study of Gemcitabine, Cisplatin +/−Veliparib in Patients With Pancreas Adenocarcinoma and a Known BRCA/PALB2 Mutation (Part I) and a Phase II Single Arm Study of Single-Agent Veliparib in Previously Treated Pancreas Adenocarcinoma	II	107	Recruiting	International (US, Canada, Israel)
**NCT02042378** [96]	A Phase 2, Open-Label Study of Rucaparib in Patients With Pancreatic Cancer and a Known Deleterious BRCA Mutation	II	100	completed	US, Israel
**NCT01489865** [66]	A Phase I/II Study of ABT-888 in Combination With 5-fluorouracil and Oxaliplatin (Modified FOLFOX-6) in Patients With Metastatic Pancreatic Cancer	I–II	48	recruiting	US
**NCT00515866** [97]	A Phase I, Open Label, Study of the Safety and Tolerability of KU-0059436 in Combination With Gemcitabine in the Treatment of Patients With Advanced Solid Tumours (Pancreatic Cancer)	I	68	completed	US, UK
**NCT01286987** [98]	A Phase 1, First in Human, Single-arm, Open-label Study of Once a Day, Orally Administered Talazoparib in Patients With Advanced or Recurrent Solid Tumours	I	74	ongoing, but not recruiting participants	US, UK
**NCT01339650** [99]	A Phase 1 Study of ABT-767 in BRCA1 or BRCA2 Mutation Carriers With Advanced Solid Tumours and in Subjects With High Grade Serous Ovarian, Fallopian Tube, or Primary Peritoneal Cancer	I	75	ongoing, but not recruiting participants	Netherlands
**NCT01989546** [100]	Pilot Trial of BMN 673, an Oral PARP Inhibitor, in Patients With Advanced Solid Tumours and Deleterious BRCA Mutations.	I–II	42	Recruiting	US
**NCT01233505** [101]	A Phase I Study of ABT-888 in Combination With Oxaliplatin and Capecitabine in Advanced Solid Tumours	I	16	terminated	US
**NCT02184195** [68]	A Phase III, Randomised, Double Blind, Placebo Controlled, Multicentre Study of Maintenance Olaparib Monotherapy in Patients With gBRCA Mutated Metastatic Pancreatic Cancer Whose Disease Has Not Progressed on First Line Platinum Based Chemotherapy (POLO)	III	145	recruiting	International
**NCT02286687**[102]	Phase II Study of the PARP Inhibitor BMN 673 (Talazoparib Tosylate) in Advanced Cancer Patients With Somatic Alterations in BRCA1/2, Mutations/Deletions in PTEN or PTEN Loss, a Homologous Recombination Defect, Mutations/Deletions in Other BRCA Pathway Genes and Germline Mutation in BRCA1/2 (Not Breast or Ovarian Cancer).	II	270	ongoing, but not recruiting participants	US
**NCT00386399a** [103]	Phase II Study of Mitomycin-C in Patients With Advanced or Recurrent Pancreatic Cancer With Mutated BRCA2 Gene	II	29	study has been withdrawn prior to enrolment	US

^a^Trial withdrawn prior to enrolment due to all 29 consented subjects testing negative for the BRCA2 mutation.

A recent whole genome sequencing study [51] supports the notion that there is also a group of patients with PDAC tumours that have a so-called *BRCA*ness phenotype which usually occurs in germline-mutated tumours, that arise from DNA repair defects due to a compromised DNA repair by homologous recombination [32].

Waddell et al. reported that germline mutations in *BRCA*1 and *BRCA*2 accounted for as few as 4 of a potential 24 patients (17%), and 4% of all patients. These data suggest that if defective DNA maintenance could be identified in addition to the mutations in the *BRCA* pathway genes, one could identify a wider group of patients with *BRCA*ness that could then be potentially treated with new therapeutics targeting that pathway [32, 51].

However, currently *BRCA*ness is a term that can apply to a variety of different mutations that might not be clinically relevant.

As the cost of WGS decreases and its use in clinical practice becomes more wide-spread, the likelihood of identifying non-significant mutations will increase. For example, it isn't known if the other 83% of potential *BRCA*-like tumours in the Waddell et al. paper [51] behave and can be targeted in the same way as germline-mutated tumours.

Therefore, if there are positive trials with new targeted therapies in germline-mutated tumours, the dilemma will emerge regarding who will be offered these agents and what would be the best predictor of response [60]. It will also be important to define the patient subgroups with non-germline *BRCA* mutations who might respond to PARP inhibitors. Other proteins involved in DDR such as ATM, ATR, CHEK1, CHEK2, DSS1, RAD51, NBS1 and those whose deficiency causes Fanconi anaemia have been assessed in preclinical studies to determine PARP inhibitor sensitivity, with early positive results in a variety of cancer cell lines [63, 64].

In ovarian cancer, the European Medicines Agency has approved the PARP inhibitor, olaparib, as the first targeted treatment for an inherited cancer disorder but also as the first *BRCA*ness-targeted therapy. It is approved in the European Union for the treatment of both germline and somatically-mutated tumours [60]. There is one clinical trial currently recruiting patients with pancreatic cancer in the US which is targeting *BRCA*ness (non-germline mutations) with olaparib [65] and this study could provide more clarity on treatment options for these subtypes of tumours.

At present it remains uncertain whether all DDR pathway defects (germline, sporadic, HRD and unstable genomes) are targets for new therapies in pancreatic cancer and this requires clinical trial assessment.

### Clinical aspects of DDR mutations in pancreatic cancer

Due to the small percentage of patients with PDAC having germline mutations, the clinical implications of these mutations are still largely unknown. There is limited data about whether germline mutations play a role in the prognosis or in potential treatment options for PDAC. Those with *BRCA*-mutation positive breast and ovarian cancers have had better outcomes with the use of platinum-based chemotherapy and PARP inhibitors in Phase III trials. These studies have led to the development of novel studies in *BRCA*-mutated pancreatic cancers with multiple different studies using PARP inhibitors as monotherapy [28], in combination with chemotherapy [66, 67], or maintenance therapy after first-line platinum chemotherapy [68].

### More favourable outcomes for patients with *BRCA*-mutated pancreatic tumours

Worse survival outcomes have been reported in *BRCA*2-mutated breast cancer (but not *BRCA*1) compared to sporadic cancers, independent of treatment, in some population-based studies, which seems to be due to adverse tumour characteristics [69]. The opposite effect was reported in patients with *BRCA*-positive epithelial ovarian cancer [70]. In the study by Golan et al. [71] of patients with pancreatic cancer with *BRCA* mutations, a slightly more favourable median all-stage OS was reported for patients with PDAC and *BRCA*1/2 mutations. Patients naïve to PARP inhibitors (*N* = 58) had a median all-stage OS of 14 months. For context, the reported OS in historical controls of patients with advanced pancreatic cancer is 4–7 months [72]. This data suggested that patients with *BRCA*1 or *BRCA*2-associated PDAC had more favourable outcomes than non-*BRCA*-associated PDAC, even amongst patients who have not received PARP inhibitors, although there are no other trials reporting similar results.

### Prognosis in patients with pancreatic cancer and *ATM* loss

In a study by Kim et al, which included 396 patients with resected pancreatic cancer, patients with *ATM* loss tended to have more vascular invasion (63.3%) and lymph node metastasis (92.2%) compared with cases without *ATM* loss (49.4% and 84.1%, respectively). However, decreased overall survival was only reported for patients who had both *ATM* loss and normal *TP53* expression, and not in patients with abnormal *TP53* expression. Nine cases which demonstrated both *ATM* loss and normal *TP53* expression had significantly reduced overall survival compared to the other 388 patients with pancreatic cancers. Following multivariable analysis, *ATM* loss, and also in combination with normal *TP53* expression, remained a significant independent predictor of decreased overall survival [53].

Waddell et al. also reported that mutations in *ATM* (and FANCM, XRCC4, and XRCC6) were linked to tumours with unstable genomes or the *BRCA*-mutational signature. Seventy-four percent of these patients had inactivation events for *TP53*, but these changes weren't correlated with outcomes in their study [51].

### Therapies producing DNA damage and targeting repair

**Figure 1:**
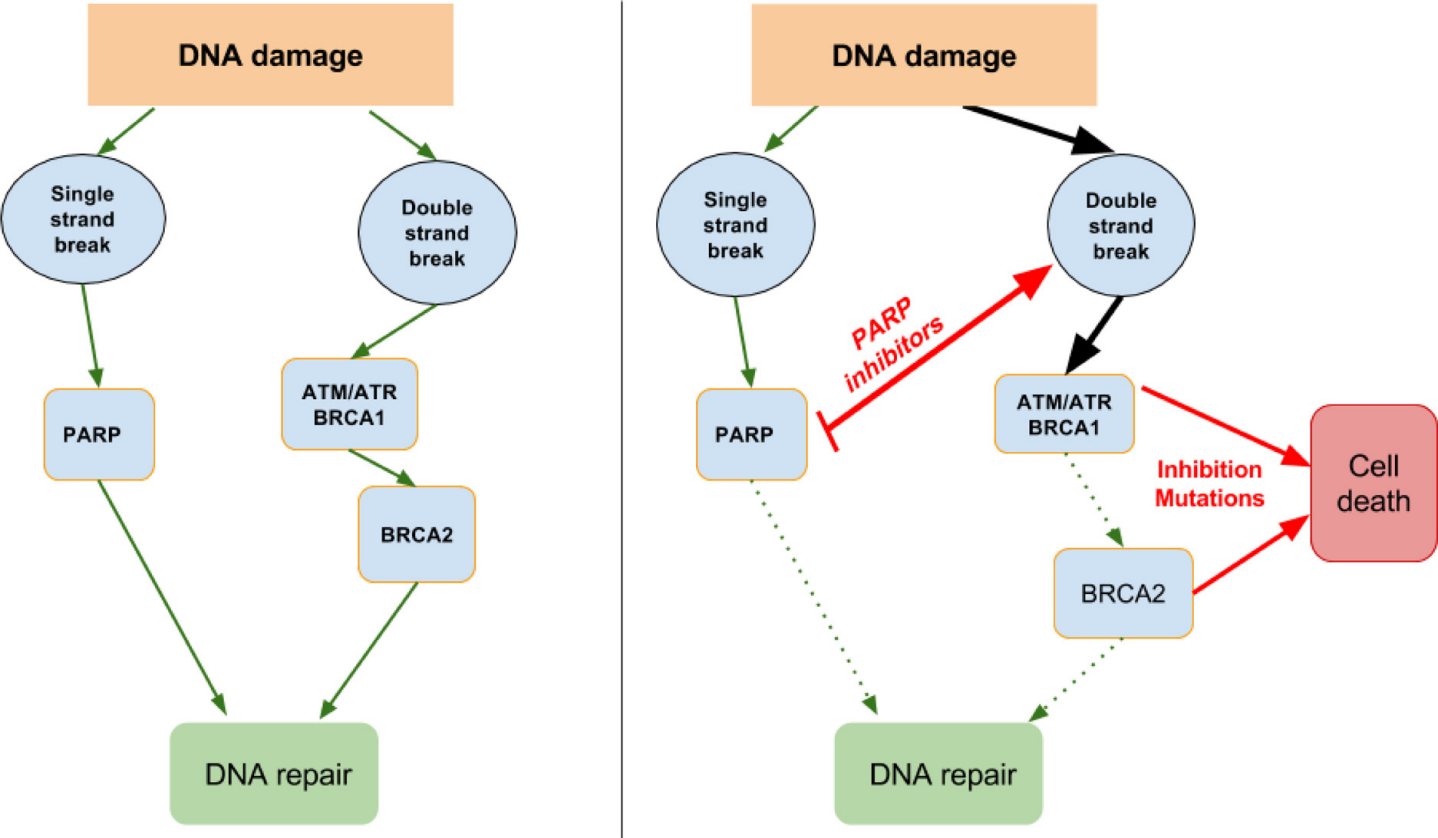
DNA damage repair and synthetic lethality Single-strand breaks need PARP for DNA repair, so inhibiting PARP will lead to double-stand breaks. In turn, double strand breaks need ATM/ATR, BRCA1 and BRCA2 for repair, so mutations or inhibition of these will lead to cell death.

Mutations and loss of DDR capacity can lead to an exploitable DDR dependency in cancers, which makes it an attractive target for therapy [73]. Cancer DDR differs from normal cells in that most cancers will have lost one or more DDR pathway functions or capability during their generation, leading to a greater dependency on the remaining pathways [74]. In precancerous cells, DDR activation represents a barrier for uncontrolled cell growth, but in cells that have progressed to form tumours, this barrier will have been removed through loss of one or more DDR capabilities. In turn, a cancer cell that harbours a DDR deficiency depends on a particular DDR target or pathway for survival and thus provides the potential for single-agent activity of an inhibitor of that target or pathway—an approach that has been described as “synthetic lethality” [63, 75] (see Figure 1).

### Platinum-based chemotherapy in patients with germline mutated PDAC

A meta-analysis has reported a small possible benefit for platinum-based therapies in the treatment of all patients with PDAC [76], and there is a suggestion that the benefit may be driven by subgroups of responders like patients with *BRCA*1/2 mutations [51]. Also FOLFIRINOX, which is the standard of care for select patients with advanced PDAC (with good performance status) contains the platinum agent oxaliplatin, and its increased use since the Conroy et al. publication [5] may provide more evidence to support a greater benefit in certain subgroups of patients with advanced PDAC.

Platinum agents are potentially more effective in patients with DDR mutations due to their cytotoxic effect by binding directly to DNA, causing crosslinking of DNA strands and thereby inducing DNA double strand breaks. If there are *BRCA*1 or *BRCA*2 mutations, the damage is not repaired effectively [77]. In pancreatic cancer, *in vitro* and *in vivo* data suggest that pancreatic cancers with *BRCA*2 mutations are more susceptible to DNA-crosslinking agents [78, 79].

In clinical trials, data on the impact of platinum agents in patients with germline-mutated pancreatic cancer are still limited. In a retrospective study by Golan et al., 58 out of 71 patients with PDAC and *BRCA*1/2 mutations received non-experimental treatments and the median OS was superior for patients with advanced disease (stage 3–4) treated with platinum vs. non-platinum chemotherapy (22 vs. 9 months) [71].

In the retrospective study conducted by Lowery et al. [77], that included patients with *BRCA*1/2 mutated PDAC, five out of six patients (83%) treated with platinum-based first-line chemotherapy for metastatic disease demonstrated a radiographic complete or partial response, which is a significantly higher response rate than historical controls, for example, in the ACCORD trial [5] published in the same year which was 31%. Interpretation of these results is limited due to small patient numbers and the varying stages of disease included [77].

In a more recent study, Aung et al. reported retrospective survival data on 57 patients with *BRCA*1/2-mutated PDAC with a median follow up of 18 months (range 2–87). Thirty three patients with advanced disease had a median OS of 9.6 months and a 2-year OS of 24%. Patients with advanced disease who received platinum compounds (oxaliplatin, cisplatin or carboplatin) (*N* = 20) during any treatment line of palliative chemotherapy, had a median OS of 15.3 months and a 2-year OS of 35%. In the other 13 patients who did not receive platinum-based chemotherapy, the median OS was 8.3 months and the 2-year OS was 0%. They concluded that in their study the OS was superior to historical controls for advanced disease, predominantly for patients treated with platinum-based chemotherapy [80].

As most of the survival data for germline-mutated patients with pancreatic cancer is retrospective, it is not possible to make definitive conclusions regarding the best therapeutic agents to prescribe for these patients. The reasons why some germline-mutated patients with pancreas cancer did not receive platinum-based chemotherapy are not stated and may relate to variations in standard of care. Some patients may have had poor performance status and so were not eligible for platinum-based chemotherapy, poor kidney function precluded its use or they were treated prior to the availability of results from the ACCORD trial [5]. There were some complete responses reported [81], and retrospective studies do present some evidence to support the use of platinum-based therapy in this subgroup of patients, although randomised prospective trials are needed.

There is a shortage of data regarding treatment with other agents such as cyclophosphamide, temozolomide and mitomycin-C that could selectively attack error-prone, homologous repair-defective cells in this subgroup of patients [71].

### Topoisomerase I inhibitors and mitomycin in patients with germline mutated PDAC

Preclinical studies have demonstrated that Fanconi anaemia-defective cancer cells are hypersensitive to the cross-linking agents mitomycin-C, cisplatin, chlorambucil, and melphalan but not to 5-fluorouracil, gemcitabine, doxorubicin, etoposide, vinblastine, or paclitaxel [79]. This suggests that varying DDR mutations in pancreatic cancer need to be characterised further prior to the development of new therapeutic possibilities [82].

In the clinical setting, topoisomerase I inhibitors and mitomycin-C may have theoretical benefit in the treatment of patients harbouring *BRCA* mutations. Vyas et al. [83] reported a great variability of clinical responses in their small study in patients with *BRCA*2-mutated PDAC. An exceptionally good response to second-line irinotecan monotherapy was seen in a patient who had 81 weeks of stable disease and another 52 weeks with irinotecan in combination with cetuximab. Two other patients who received irinotecan as third-line treatment in the combination regimen, 5-fluorouracil, leucovorin and irinotecan (FOLFIRI) had a duration of response of 16 and 56 weeks. Two other patients were treated with mitomycin-C monotherapy in a third-line setting and had durations of response of 9 and 12 weeks, respectively.

Due to the small number of patients and the variability of treatment responses, the role of topoisomerase I inhibitors and mitomycin-C use in patients with germline-mutated PDAC remains to be clearly defined [83]. The recent phase III NAPOLI-1 trial reported that nanoliposomal irinotecan in combination with 5-fluorouracil and folinic acid resulted in a superior overall survival in the second-line treatment setting compared to 5-fluorouracil and folinic acid alone in patients with advanced PDAC. This trial provides a new second-line option for all pancreatic cancers and may possibly provide new information about the efficacy of topoisomerase I inhibitors also in germline-mutated tumours [84].

The use of combinations of DDR agents with DNA-damage-inducing chemotherapies may be more toxic than other regimens due to overlapping toxicities, specifically gastrointestinal and bone marrow systems. This phenomenon has resulted in a number of clinical trials being terminated early due to unacceptable adverse events [73]. A recent randomised multi-centre phase I/II study in patients with advanced pancreatic cancer (NCT01296763), of irinotecan, cisplatin and mitomycin-C (ICM) with or without olaparib [85] consisted of 3 ± 1 different DDR-targeting agents administered together. This trial did not progress to phase II, and the results are only available on-line in clinicaltrials.gov. From the data presented, there were serious adverse events reported in 67–80% of patients and adverse events in 100% of patients associated with different dose levels. The most common adverse events were anaemia and nausea.

### PARP inhibitors in patients with germline mutated or sporadic PDAC

Inhibiting PARP in cells causes the persistence of DNA lesions normally repaired by homologous recombination [86], which in turn leads to the induction of double-strand breaks after stalling and collapse of the DNA replication forks. Tumours in which there is a defect in homologous DNA repair (and thus defective repair of double-strand breaks) seem to be susceptible to PARP inhibitor therapy [28]. Tumours of germline *BRCA* mutation carriers lack wild-type functional copies of *BRCA*1 or *BRCA*2, but normal tissues retain a single wild-type copy of the relevant gene [73], making treatment with PARP inhibitors highly tumour-specific, and less toxic [86], as the tumours in *BRCA*-mutated patients are defective in homologous recombination [87]. This difference between tumour and normal cells is exploited by PARP inhibitors and this provides a selective therapeutic window [73].

Early clinical trials in patients with *BRCA*-mutated PDAC have reported positive responses to PARP inhibitors [28, 88]. A phase II trial of olaparib monotherapy for patients with germline *BRCA*1/2-mutated advanced cancer included 23 patients with PDAC and reported a response in 5 patients (21.7%); complete response in 1 (4.3%), and partial response (PR) in 4 (17.4%). Stable disease (SD) lasting ≥ 8 weeks was reported in 8 (34.8%); 36.4 % were progression-free at 6 months, and 40.9 % were alive at 12 months [28].

Another phase II clinical trial that evaluated the efficacy of single agent Veliparib in patients with *BRCA* or *PALB2*-mutated pancreatic cancer after first or second-line chemotherapy enrolled 16 patients with advanced disease and reported 1 unconfirmed PR, 4 patients with SD, 10 with progressive disease and 1 was not evaluable. Median progression-free survival was 52 days (range 12 to 423), and 4 patients (25%) remained on study with SD for ≥ 4months. The study concluded that there was some single-agent activity of Veliparib in patients with previously treated PDAC [89].

A Phase IB trial of the PARP inhibitor, veliparib, in combination with cisplatin and gemcitabine in patients with *BRCA* or *PALB2*-mutated pancreas adenocarcinoma reported 5 (56%) partial responses (PR), and 4 (44%) with stable disease in 9 patients who were *BRCA*-mutated [90]. A phase II trial is currently recruiting examining this combination [67].

A phase III randomised multicentre study of maintenance olaparib/placebo monotherapy in patients with germline *BRCA*1/2-mutated metastatic PDAC (POLO clinicaltrials.gov: NCT02184195) [68] who have stable disease after at least 16 weeks of platinum-based chemotherapy is currently ongoing and may provide valuable information, particularly regarding progression-free survival of these patients.

### ATM and ATR targeted agents in patients with germline mutated PDAC

A wide variety of DNA lesions [91] and DNA damage caused by radiotherapy or chemotherapy leads to activation of the DNA-damage response, involving activation of cell cycle checkpoints and DNA repair. Ataxia-telangiectasia mutated and ATR (ataxia telangiectasia and Rad3-related protein) are the two key kinases involved in DNA signalling and they have the ability to detect single-strand and double-strand DNA breaks [92]. They are involved in mediating the cellular response to double-strand breaks and replication stress [64], and thus provide a new potential target in the DDR pathway. It has been reported that deficiency in ATM and reduction of ATR kinase activity cause defects in homologous recombination and sensitivity to PARP inhibition [60].

The ATR inhibitor VE-822 (Vertex Pharmaceuticals, USA), has been reported to radio-sensitise p53-mutated pancreatic cancer cell lines *in vitro* and in xenograft models of human pancreatic cancer and further increases the growth delay induced by ionising radiation (IR) combined with gemcitabine. Importantly, VE-822 did not cause extra toxicity in normal tissues or cells and was well tolerated in mice [93, 94].

Activity of a second ATR inhibitor, AZD6738 (AstraZeneca, UK), is currently being examined in a Phase I clinical trials for advanced cancer. Increased tumour growth inhibition has been reported when this has been combined with carboplatin or radiotherapy *in vivo*, and single-agent anti-tumour activity has been seen in ATM-deficient but not ATM-proficient xenograft models [93].

The first ATM inhibitor, utilised in *in vivo* studies was KU59403 (KuDOS Pharmaceuticals, now AstraZeneca, UK), and although it was not cytotoxic to human cancer cell lines, it significantly increased the cytotoxicity of topoisomerase I and II agents: camptothecin, etoposide and doxorubicin [93].

Another ATM inhibitor, AZD0156 (AstraZeneca, UK), is currently being investigated in a Phase I clinical trial for advanced solid tumours as monotherapy or in combination with either olaparib, cytotoxic chemotherapies, or novel anti-cancer agents to assess safety, tolerability and anticancer activity of these treatments [95].

Table 3 provides details on current trials recruiting patients with advanced cancers (including PDAC) where ATM or ATR inhibitors are administered as mono- or combination therapy. Possible synthetic lethal interactions may be produced in patients with pancreatic cancer using the new targeted therapies, ATM or ATR inhibitors, as ATM and ATR are key participants in DNA repair [54].

**Table 3:** Current trials utilising ATM or ATR inhibitors in patients with advanced malignancies (including pancreatic cancer) registered on Clinicaltrials.gov

Trial ID		Phase	Number of patients	Status of trial	Countries involved
**NCT02588105** [95]	A Phase I, Open-Label Study to Assess the Safety, Tolerability, Pharmacokinetics and Preliminary Efficacy of Ascending Doses of AZD0156 Monotherapy or in Combination With Either Cytotoxic Chemotherapies or Novel Anti-Cancer Agents in Patients With Advanced Malignancies	I	225	recruiting	International
**NCT02223923** [104]	A Phase I Study to Assess the Tolerability, Safety and Biological Effects of ATR Inhibitor (AZD6738) as a Single Agent and in Combination With Palliative Radiation Therapy in Patients With Solid Tumours	I	100	recruiting	UK
**NCT02264678** [105]	A Modular Phase I, Open-Label, Multicentre Study to Assess the Safety, Tolerability, Pharmacokinetics and Preliminary Anti-tumour Activity of AZD6738 in Combination With Cytotoxic Chemotherapy and/or DNA Damage Repair/Novel Anti-cancer Agents in Patients With Advanced Solid Malignancies	I	114	recruiting	International
**NCT02595931** [106]	Phase I Clinical Trial of VX-970 in Combination With the Topoisomerase I Inhibitor Irinotecan in Patients With Advanced Solid Tumours	I	51	recruiting	US
**NCT02723864** [107]	Phase I Study of Veliparib (ABT-888), an Oral PARP Inhibitor, and VX-970, an ATR Inhibitor, in Combination With Cisplatin in Patients With Refractory Solid Tumours	I	60	recruiting	
**NCT02630199** [108]	Phase I, Open-Label Study of AZD6738, DNA Damage Repair/Novel Anti-cancer Agent, in Combination With Paclitaxel, in Refractory Cancer	I	21	recruiting	Korea

## DISCUSSION

In this review, published data on outcomes and treatment of patients with germline-mutated pancreatic cancer was interrogated.

Pancreatic cancer is a deadly disease with short overall survival and the need for new treatment options is crucial. The published data suggests that depending on the family history of cancer, there may be varying levels of both germline and sporadic mutations in patients with pancreatic cancer. Currently, somatic mutations have not been correlated with family history, and thus differences in prevalence depending on cancer risk in a family are unknown. Depending on family history the BRCA1/2 mutation rates vary between 5–10% [33, 52] and *ATM* mutations in the same range [53, 54], while *CDKN2A* mutation levels range from half of that to 3 times higher depending on the depth of sequencing [35, 51], and *TP53* mutations are identified in up to 3 out of 4 patients [51]. Also, smaller numbers of mismatch repair [21], *PALLD* [35] and *FANCC* [56] mutations have been identified. Many of these mutations are not the more common *BRCA*1 and *BRCA*2 variants, indicating other possible targets for future treatment in these patients.

Another issue is the role that family history has on the risk of developing pancreatic cancer, and if this could be used as a selection method for mutation screening. At least one study [9] has been reported where the new NCCN guidelines have retrospectively been applied, and all patients were found to have *BRCA*1/*BRCA*2 mutations. Unfortunately, only one other study has adopted this approach using the relevant NCCN guidelines at that time, and reported contrasting results [33]. However, the guidelines applied at that time were different. At the moment, international and national FPC databases still recruit high risk patients based primarily on family history of cancer (pancreatic, breast, ovarian, melanoma, colorectal etc.) and then screen them for mutational status. Reverse approach screening of all patients with PDAC for mutations and then assessment of their relatives may be an alternative option, but may not be economically feasible.

Data herein also demonstrate that the mutational prevalence might differ between countries [35] and ethnic groups [43]. At the moment the very high risk groups for pancreatic cancer are still patients of AJ descent, as approximately 1.1% of the Jewish population carry a *BRCA*1 founder mutation and 1.1% carry a *BRCA*2 founder mutation [43], and families with ≥ 3 first- or second-degree relatives with PDAC.

Other high-risk subgroups based on genetic information remain somewhat uncertain. For *BRCA* mutations, the current NCCN guidelines (version 2.2016) give good guidance on identification of mutated patients. However, for other mutations, data is lacking. There is currently evidence to support discussion of mutational screening in patients with a family history of PDAC, in those with high prevalence of breast or ovarian cancer in the family, colorectal cancer in first-degree relatives and in those with young onset of PDAC.

There is a need to investigate the *BRCA*ness concept further in patients with pancreatic cancer. At the moment only two whole genome studies have provided information about possible *BRCA*ness features of tumours that are not germline-mutated. There is currently no prospective data correlating with clinical characteristics of these patients, although one clinical trial is currently recruiting patients with these mutations who are treated with PARP-inhibitors [65].

Currently the effectiveness of DNA-damaging cytotoxic chemotherapy, like platinum agents in patients with germline-mutated PDAC is still theoretical, as most of the studies have been retrospective and thus there may be survivor bias. However, based on these retrospective studies, platinum-based chemotherapy has the highest probability of eliciting better survival outcomes and is currently the recommended treatment strategy in these patients until further prospective trials are reported.

There is the need for novel prospective trials to include patients with germline mutations and PDAC and those with *BRCA*ness properties, as these, if successful, could potentially result in better therapeutic approaches for these patients, more effective treatment outcomes, longer survival and subsequently replace current standard of care.

There are still unanswered questions relating to the most ideal therapy for patients with *BRCA*ness properties. One of these is whether *BRCA*ness, *BRCA*-like or HRD tumours can be treated similarly to germline-mutated patients. The best predictor of a favourable response to a drug that targets DDR is still unknown [60].

Whole genome sequencing has provided strong evidence that there are multiple other changes in these tumours in addition to germline mutations, although it is not known whether these changes are targetable by the same novel therapeutics, and so future studies should attempt to address this dilemma in clinical trials.

The PARP and ATM/ATR inhibitors are currently the most promising novel agents undergoing investigation in these solid tumours, as DDR seems to be affected in most of these germline-mutated or *BRCA*-like cancers. It may be that aligning DNA damage-inducing chemotherapy to the specific inhibition of a DDR protein that repairs that damage [73] is the best therapeutic strategy. Further research into different combinations of cytotoxic and targeted therapies is obligatory in pancreatic ductal adenocarcinoma and in those displaying *BRCA*ness properties. At this time, treatment with platinum agents is the standard of care for patients with germline-mutated PDAC and discussions about genomic testing should be conducted in patients with a strong family history or young onset of PDAC. All appropriate patients with germline and/or somatic mutations and their relatives should be directed to enter clinical or screening trials when possible.
